# Liqui-Mass Technology as a Novel Tool to Produce Sustained Release Liqui-Tablet Made from Liqui-Pellets

**DOI:** 10.3390/pharmaceutics13071049

**Published:** 2021-07-09

**Authors:** Matthew Lam, Nour Nashed, Ali Nokhodchi

**Affiliations:** Pharmaceutics Research Laboratory, Arundel Building, School of Life Sciences, University of Sussex, Brighton BN1 9QJ, UK; N.Nashed@sussex.ac.uk (N.N.); A.Nokhodchi@sussex.ac.uk (A.N.)

**Keywords:** liqui-tablet, liqui-mass technology, liqui-pellet technology, sustained release, eudragit rs po, kinetic model, matrix pellet

## Abstract

The Liqui-Mass technology (also known as Liqui-Pellet technology) has shown promising results in terms of enhancing the drug release rate of water insoluble drugs in a simplistic approach. However, there is no current study on sustained-release formulation using the Liqui-Mass technology. In this study, an attempt was made to produce a sustained-release Liqui-Tablet for the first time using a matrix-based approach. The non-volatile co-solvent used in the investigation included Tween 80, Tween 20 and Kolliphor EL. The production of sustained-release propranolol hydrochloride Liqui-Tablet was successful, and data from the saturation solubility test and dissolution test did not show much difference among the mentioned non-volatile co-solvent. The best Liqui-Tablet formulation took 24 h for drug release to reach at around 100%. There seemed to be a synergistic retarding drug release effect when a non-volatile co-solvent and Eudragit RS PO were used together. The increase of Eudragit RS PO concentration increased the retardant effect. Kinetic drug release analysis suggests that the best formulation followed the Higuchi model. The flowability of pre-compressed Liqui-Tablet pellets had no issues and its size distribution was narrow. Liqui-Tablet was generally robust and most formulations passed the friability test. The study revealed that Liqui-Mass technology can be employed to sustain drug release.

## 1. Introduction

Liqui-Mass technology, or sometimes referred to as Liqui-Pellet technology, is a recently developed oral drug delivery system, which was first filed for a patent in 2018 and published internationally in 2020 [1]. It was developed in response to bringing the concepts from liquisolid technology into a commercially feasible direction. Experimental data in terms of enhanced dissolution rate and manufacturability have been shown to be very promising [2,3,4,5,6]. The idea of an active pharmaceutical ingredient (API) being in a solubilised state in a solid matrix carrier system has not yet been seen in the current market according to the authors’ current knowledge. This is due to major technical issues with liquisolid technology, which are still persisting after more than two decades. Such drawbacks include issues with flow property, compressibility and inability to exist as a reasonably sized dosage form when high-dose formulation is required [7,8,9,10]. In terms of flow property, the incorporation of liquid medication in dry powder excipients is problematic because it makes the powder cohesive, giving rise to issue concerning flowability. This in turn creates an issue with attaining uniform feed and reproducible filling [11], which is a major concern when considering large-scale production. High-dose drug typically needs more liquid vehicle, which in turns need more excipients powder to improve formulation flowability. However, this would result in an end product being too large for actual use in patients. With the development of Liqui-Mass technology, such an issue is resolved, leading to a presumption that Liqui-Pellet and Liqui-Tablet are highly feasible in a commercial sense [2,3,4,5,6].

Since Liqui-Mass technology is new, there may be initial confusion about whether Liqui-Mass and liquisolid technologies are the same, as shown in the commentary by Pezzini et al. [12]. Lam et al. [13] responded to this commentary by explaining in detail the differences. In brief, Liqui-Pellet and liquisolid technologies share some similarities, but there are crucial differences separating them. Both contain API that is solubilised in a liquid vehicle and incorporated into carrier and coating material; however, the admixture for liquisolid formulation is under liquisolid system, which is an extensively used and defined term in liquisolid technology. The term “liquisolid systems” refers to powdered forms of liquid medications formulated by converting liquid lipophilic drugs, or drug suspensions or solutions of water-insoluble solid drugs in suitable non-volatile solvent systems, into “dry” (i.e., dry-looking), nonadherent, free-flowing and readily compressible powder admixtures by blending with a selected carrier and coating materials [14]. Notice such a system is a dry-looking and free-flowable powder admixture. Liqui-Pellet cannot be described as a liquisolid formulation, as it does not fall under the liquisolid system. The API and excipients admixture are not dry-looking and nonadherent but are in fact in a form of wet mass even before incorporating granulating liquid, which will eventually get removed via evaporation. Such a system is termed a Liqui-Mass system. This crucial difference between liquisolid technology and Liqui-Mass technology makes Liqui-Pellets and Liqui-Tablets much more versatile with a high liquid load factor and having remarkable dissolution profile, but most importantly, makes them feasible in a commercial sense.

Currently, the Liqui-Mass technology has only been employed to enhance the dissolution of poorly water-soluble drugs [2,3,4,5,6], and a prolonged-release formulation is yet to be explored. This investigation aimed to produce a prolonged-release Liqui-Tablet for the first time. On looking at the classical liquisolid technology, there are limited studies on sustained-release liquisolid formulation. It seems that the concept of solubilised API in solid powder admixture can hold some key advantages, notably the zero-order drug release kinetic [15,16,17]. Zero-order release kinetic can be considered very useful in sustained release formulation in terms of clinical aspect, safety and predictability in regard to pharmacokinetics.

The prolonged-release formulation is a growing area of research due to the benefits it can offer to patients, healthcare staff and pharmaceutical companies. It offers a more robust and consistent drug release than conventional pills, which means better control of drug plasma level. The sustained-release formulation can also aid patient compliance, reduce the burden on healthcare professionals and can possibly extend the product’s patent life [18,19]. Therefore, there is an incentive to explore prolonged-release oral dosage forms using Liqui-Mass technology. Propranolol hydrochloride is a non-selective beta-adrenergic receptor antagonist that is widely used for treating hypertension and arrhythmias [20]. It was the chosen API in this study to investigate the feasibility of producing a prolong-release Liqui-Tablet. Propranolol has a short half-life of around 3 h and good permeability and solubility (BCS Class 1), thus making it a suitable drug model for sustained-release formulations. An API with good water solubility is a good candidate for a sustained-release formulation.

Although Liqui-Mass technology has only been applied to enhance drug release, it is inherently suitable for sustained release. The microcrystalline cellulose (MCC), which is the gold standard diluent for making pellets via the extrusion-spheronization method, is known to be virtually non-disintegrating and suitable for sustained drug release [21].

**Figure 1:**
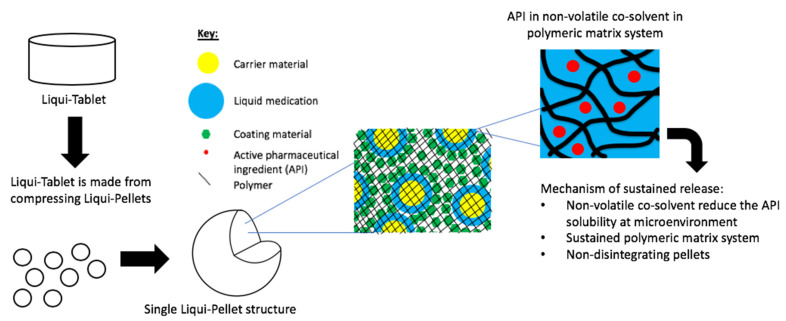
General structure of sustained-release Liqui-Tablet.

The investigation aimed to produce a sustained release propranolol Liqui-Tablet through utilizing the non-disintegrating MCC-based pellets, retarding agent (Eudragits RS PO) and liquid vehicles. This sustained-release Liqui-Tablet used a matrix system approach, thus omitting the commonly used polymeric film coating. This simplified the manufacturing process and removed the issue of film coating rupturing on compression during the production of the pellet-based tablet. The general structure of sustained release Liqui-Tablet can be seen in Figure 1. The non-volatile co-solvent reduced the API solubility, and the retardant polymer was within the whole pellet structure, which was compressed into Liqui-Tablet.

## 2. Materials and Methods

### 2.1. Chemicals

Propranolol hydrochloride (TCI, Tokyo, Japan); Avicel PH-102 (FMC Corp., Philadelphia, PA, USA); Aerosil 300 (Evonik Industries AG, Hanau, Germany); propylene glycol (Acros Organic, Fisher Scintific, Loughborough, UK); Tween 80 (Acros Organic, Fisher Scintific, Loughborough, UK); Tween 20 (Acros, Geel, Belgium); Kolliphor EL (BASF SE, Ludwigshafen, Germany) and Eudragit RS PO (Evonik, Essen, Germany). All other reagents and solvent were of analytical grades.

### 2.2. Saturation Solubility Test

The liquid vehicles used in the saturation solubility tests of propranolol hydrochloride were Tween 80, Tween 20 and Kolliphor EL. A saturation solubility test of propranolol hydrochloride in deionised water was also carried out. The liquid vehicles were chosen based on the Javadzadeh et al. study [17], where they made sustained release liquisolid formulations containing propranolol hydrochloride. The test was prepared by adding propranolol hydrochloride in excess in a vial containing 10 mL of specified liquid, which was subjected to a constant condition of 37 °C and shaking agitation speed of 40 rpm for 24 h in a bath shaker. The filtration of the supernatant was carried out through a pre-heated filter of pore size 0.22 μm (Merck Millipore Ltd., Carrigtwohill, Ireland), which was then diluted with deionised water and analysed using a UV/ vis spectrophotometer (Biowave II, Biochrom Ltd., Cambrige, UK) at a wavelength of 288 nm. The absorbance obtained was used to determine the concentration of propranolol hydrochloride. Each test was carried out in triplicates and the mean and standard deviation are reported.

### 2.3. Production of Propranolol Liqui-Tablet

The Liqui-Tablet formulations were made by compacting Liqui-Pellets under specified compression force using a manual tablet press machine (Compaction model MTCM-I, Globe Pharma, New Brunswick, NJ, USA) to produce a standard convex tablet with a diameter of 10 mm. All Liqui-Pellet formulations were produced in this manner, with variation in the parameters such as type of liquid vehicle, the amount of liquid vehicle, water content, the presence or absence of the retarding agent (Eudragit RS PO) and ratio of a binary mixture of carriers, as shown in Table 1. Propranolol hydrochloride was mixed in a specified non-volatile co-solvent using a pestle and mortar. This liquid medication was then blended into a specified carrier or binary carrier material. The mixture of liquid medication and carrier was blended for 2 min at a constant rate of 125 rpm (Caleva Multitab, Caleva Process Solutions Ltd., Dorset, UK). A specified amount of deionized water, which acted as a granulating liquid, was incorporated slowly into the admixture to achieve an acceptable rheological property for extrusion (Caleva Multitab, Caleva Process Solutions Ltd., Dorset, UK). The admixture with water was blended for 5 min, then Aerosil 300 (coating material) was added and further blended for another 5 min before extrusion. After the specified sample was extruded, the extrudates were spheronized at an almost constant rotation at 4000 rpm. This spheronization process was reduced to 2000 rpm if the extrudate was likely to agglomerate. The spheronization time varied depending on the extrudates’ plastic property to avoid agglomeration. The spheroids were then left to dry overnight using an oven set at a constant temperature of 40 °C.

In order to compare the performance of tablets made from Liqui-Pellets with physical mixture counterpart formulations, a physical mixture tablet was prepared in a similar manner as the Liqui-Tablet but with liquid vehicle omitted. All formulations’ carrier-to-coating-material ratios were kept constant at 20:1.

### 2.4. Flowability Test on Pre-Compressed Formulations

All pre-compressed tablet formulations in the form of pellets were subjected to various flow property tests. The flow property tests included the typical angle of repose, Carr’s compressibility index and a simple flow rate test where samples were let to flow through an orifice, and mass per second was recorded. These tests were done in triplicates to increase data reliability.

### 2.5. Particle Size Analysis Using the Sieve Method

The size distribution of pre-compressed pellets was determined using the mechanical shaker and sieve method (Test sieve, Retsch, Haan, Germany). Each formulation of weight around 5 g was subjected to the mechanical shaker and sieve particle size analysis with an amplitude of 50 for 1 min and an amplitude of 40 for a further 9 min. The higher amplitude was only used to speed up the initial sieving process but could cause damage to the pellets; therefore, the remaining sieving process was at a lower amplitude. The size of the sieve used was 2000, 1000, 850, 500 and 250 µm sieves. The pellets yield in these sieves were then determined and calculated as the percentage of total pellet weight.

### 2.6. Friability and Tablet Hardness Test

All formulations in tablet form were subjected to a friability test. Ten tablets for each formulation were placed in a friabilator (D-63150, Erweka, Langen, Germany), which rotated for 4 min at a constant rate of 25 rpm. Samples that showed fracture or had a percentage weight loss of more than 1% were considered not robust enough and thus failed the test.

Tablet hardness test was carried out on each formulation using the tablet hardness tester (TBH 125, Erweka, Germany). In this test, the amount of force (in Newton) required to fracture the tablet was recorded. This was tested on 3 tablets made from each formulation, and the mean was calculated.

### 2.7. In Vitro Drug Release Test

Each Liqui-Tablet and physical mixture tablet formulations contained 80 mg of propranolol hydrochloride. These tablets were subjected to a dissolution test in accordance with the USP paddle method (708-DS Dissolution Apparatus and Cary 60 UV-Vis, Agilent Technologies, Santa Clara, CA, USA) at 50 rpm as recommended in the FDA draft guidance on propranolol hydrochloride [22]. The tablets were placed in the dissolution medium containing hydrochloric acid buffer solution of pH 1.2 for 2 h, followed by changing the pH of the dissolution medium from 1.2 to 7.4 by adding concentrated phosphate buffer solution (for 22 h). The pH 1.2 and 7.4 simulated gastric fluid and intestinal fluid (without enzymes), respectively. The temperature of the dissolution medium was kept constant at 37.3 ± 0.5 °C. The absorbance readings were taken at a wavelength of 288 nm at a time interval of 15 min unitl 2 h, then every 2 h until 24 h. This wavelength accords with the reference used in *Clarke’s Analysis of Drugs and Poisons* [23] and published articles [17,24], which also state that there is no shift in absorbance at 288 nm in acidic or alkaline conditions.

### 2.8. Dissolution Profile Comparison via Model-Independent Analysis

Where possible, Equation (1) (difference factor, *f*_1_) and Equation (2) (similarity factor, *f*_2_) were used to compare the dissolution profile of different formulations [25]. Both equations have been recommended by the US FDA (Food and Drug Administration) [26] and are implemented by the FDA in various guidance documents [27,28]. An *f*_1_ value between 0–15 and *f*_2_ value between 50–100 implies the equivalence of the two dissolution profiles [29]. Details of the equations can be found in various reports in the literature [26,30,31,32]. In general, in the following equations, *n* represents the number of dissolution sample times and R_*t*_ (reference) and T_*t*_ (test) represent the mean percentage of drug dissolved at each time point (*t*).

*f*_1 = {[Σ*t* = 1_^*n*|^R_*t*_ − T_*t*_^|^_]/[Σ*t* = 1_^*n*^ R_*t*]} 100_ (1)

*f*_2 = 50 log {[1 + (1/*n*) Σ*t* = 1_^*n*^ (R_*t* −_ T_*t*_)^2^_]_^−0.5^_100}_ (2)

### 2.9. Kinetic Model Analysis of Drug Release

The drug release data obtained for each formulation were applied to different drug release kinetics models. The mathematical models that were used included zero-order, first-order, Higuchi and Korsmeyer–Peppas model. A zero-order release model describes a system where a drug release rate is constant in such a way that it is independent of its concentration [33,34]. The data from the cumulative drug release can be plotted against time [33,34,35]. A first-order release model describes a system where a drug release rate is dependent on its concentration and can be obtained by plotting logarithm percentage release of remaining drug vs the time [33,34]. The Higuchi model suggests that drug release from an insoluble matrix is directly proportional to the square root of time and is based on Fick’s law of diffusion. The plot of cumulative percentage of drug release against the square root of time should be linear if drug release is a controlled release [36]. By using the Korsemeyer–Peppas mathematical model, it is possible to study the drug release mechanism using the *n* value, which is the diffusional exponent or drug release exponent. The most appropriate kinetic model for a formulation is based on the highest square of correlation coefficient represented as *R*^2^ value and the lowest mean percentage error [34].

## 3. Results and Discussion

### 3.1. Saturation Solubility Test

The data from the saturation solubility test in Table 2 clearly show that propranolol hydrochloride is less soluble in non-volatile liquid vehicle than in water (~29 mg/mL). The solubility of propranolol in Tween 80 (1.89 mg/mL), Tween 20 (1.86 mg/mL) and Kolliphor EL (1.32 mg/mL) are very similar. In a study by Javadzadeh et al. [17], other liquid vehicles such as propylene glycol, PEG 400, PEG 200 and glycerine were used; however, the results showed propranolol hydrochloride had the lowest solubility in Tween 80.

### 3.2. Production of Propranolol Liqui-Tablet

All formulations were successfully made into Liqui-Tablet, indicating that it is possible to incorporate Eudragit RS PO into the Liqui-Mass system to produce pellets via extrusion-spheronization. In addition, these pellets can be compressed into tablet dosage form, producing Liqui-Tablet. It was observed that by increasing the amount of Eudragit RS PO there is less amount of granulating liquid (deionised water) required to produce the pellets, as shown in Table 1. This is evident through formulation F-6 (13.7% Eudragit RS PO per dosage form) requiring more than 3 times the granulating liquid as F-8 (41% Eudragit RS PO per dosage form) to successfully produce pellets.

### 3.3. Flowability Test on Pre-Compressed Propranolol Liqui-Tablet

There is no issue regarding the flow property of all pre-compressed Liqui-Tablet formulations as shown in Table 3. The inference from the angle of repose shows excellent flowability for all formulations, and the inference from Carr’s compressibility index shows mainly good flowability. This is ideal in terms of smooth commercial manufacturing and quality control test, such as the content uniformity test. The smooth flow has been observed in other studies on Liqui-Mass technology [2,3,4,5,6]; therefore, it is claimed that Liqui-Mass technology has overcome the poor flowability problem that is seen extensively in liquisolid technology.

### 3.4. Particle Size Analysis Using the Sieve Method

**Figure 2:**
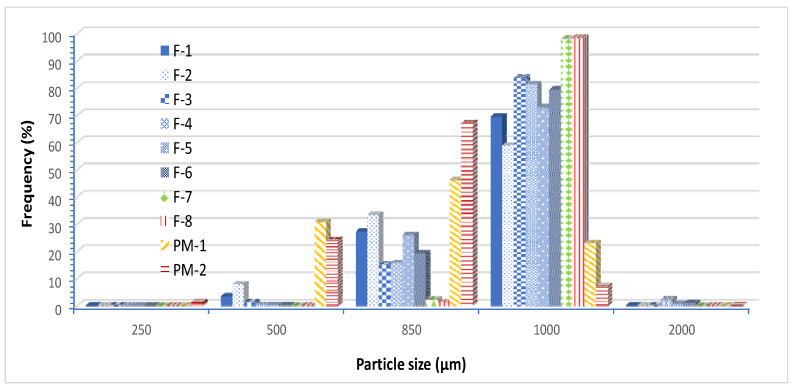
Particle size distribution of all formulations.

Data from particle size analysis in Figure 2 show that all pre-compressed Liqui-Tablet formulations have a narrow size distribution. All pre-compressed Liqui-Tablet fall mainly under 1000 µm sieve, whereas physical mixture is mainly under 850 µm sieve. The data seem to show that the presence of liquid vehicle may be contributing to the increased pellet size. Perhaps the cohesive force due to wet liquid vehicle or the increase in the plastic property of extrudate influences the pellet size during the spheronization process.

### 3.5. Tablet Friability and Hardness Test

All formulations passed the friability test except for the physical mixture tablets and F-5 (Table 4). Formulation F-5 has the highest amount of liquid vehicle, which may have contributed to its reduction in robustness; hence, F-5 fractured. Other than formulation F-5, all Liqui-Tablet formulations show good robustness, which is essential for potential upscale production.

It was observed that weight loss was negligible for most Liqui-Tablet formulations. The liquid vehicle could be increasing the Liqui-Tablet plasticity due to the plasticising effect, which can increase pellet resistance to friability.

In terms of the data obtained from tablet hardness test (Table 4), liquid vehicle does not have negative impact but instead increased the compactability of Liqui-Tablet, which can be considered a major advantage. It can be generally seen that Liqui-Tablet, which contains liquid vehicle, is about 5 times harder than its corresponding physical mixture tablet. The *t*-test show that all Liqui-Tablet formulations have a significantly higher hardness than the physical mixture tablet (*p* < 0.05). This shows that liquid vehicle can in fact improve the tablet harndess.

### 3.6. In Vitro Drug Release Test

**Figure 3:**
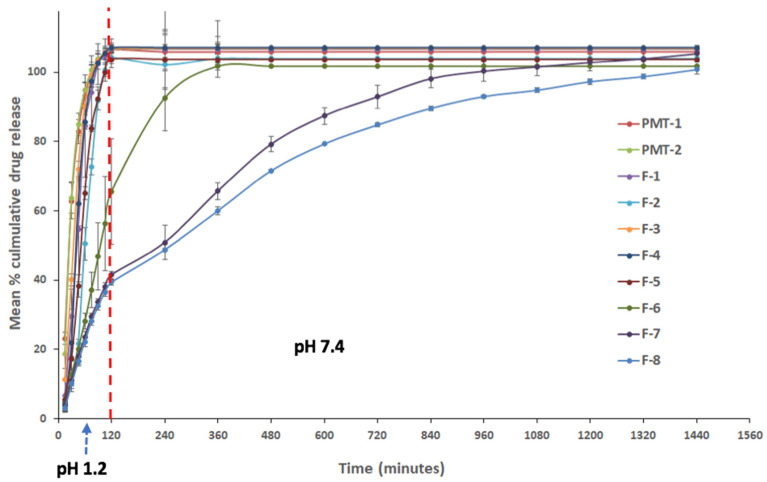
Dissolution profiles of all formulations at pH 1.2 for the first 2 h, then pH 7.4 up to 24 h.

Propranolol hydrochloride sustained-release matrix-based Liqui-Tablet was successfully produced with good extended-release, as shown in formulation F-8 in Figure 3. Formulation F-8 reached around 100% drug release after about 24 h. Such a result is ideal for a sustained-release oral dosage form since extended-release drugs are typically designed to be taken once a day by patients.

All Liqui-Tablet formulations except for F-6, F-7 and F-8 showed around 100% drug release within 2 h at pH 1.2. This is expected, as there was no Eudragit RS PO in the formulations (F-1 to F-5) to retard the drug release rate.

When comparing the dissolution profile of the physical mixture with and without Eudragit RS PO, they both show equivalence. This is supported by the model-independent analysis using the similarity and difference factor. The *f*_1_ is 3.50 and *f*_2_ is 79.65. It was initially assumed that PMT-2, which contains Eudragit RS PO, would show a slower drug release rate than PMT-1 (without Eudragit RS PO); however, the result shows no differences. This suggests that propranolol hydrochloride solubility could have rendered the Eudragit RS PO retarding effect to be less effective. It should also be noted that due to fast drug release, the time point taken for this analysis was limited.

When investigating the effect of liquid vehicles on retarding drug release rate by comparing F-1 to F-3 with PMP-1, there seems to be a significant influence. In comparing F-1 to F-3 with PMT-1, all difference factors were above 15, and all similarity factors were below 50, indicating these dissolution profiles were not similar and that the liquid vehicles were slowing down the drug release rate. Studies from Javadzadeh et al. [17] also observed that liquid vehicle (Tween 80) had a retarding effect on propranolol hydrochloride liquisolid compact. Formulations F-1 to F-3 are identical except for the liquid vehicle used. Formulation F-2 (containing Tween 20) had the largest *f*_1_ and smallest *f*_2_ (*f*_1_ = 204 and *f*_2_ = 19) compared with F-1 (containing Tween 80, *f*_1_ = 49 and *f*_2_ = 34) and F-3 (containing Kolliphor EL, *f*_1_ = 23 and *f*_2_ = 45), suggesting that Tween 20 was the most suitable choice of liquid vehicle in this study.

The combination of liquid vehicle and Eudragit RS PO in the formulation seems to have a synergistic retarding effect on Liqui-Tablet, which can be seen when comparing F-6 with PMP-2, where both formulations are identical except F-6 contains a liquid vehicle. Both the liquid vehicle and retardant seem to be an essential combination for a noticeable retarding drug release performance.

The dissolution test data show that when Eudragit RS PO was incorporated in Liqui-Tablet and its concentration increased, the drug release rate can be retarded markedly. Formulation F-7 (Eudragit RS PO 27.36% *w*/*w*) has a slower drug release rate than F-6 (Eudragit RS PO 13.68% *w*/*w*). F-7 starts reaching around 100% drug release after around 1320 min (or 22 h), whereas in the case of F-6, 100% drug release was reached within 360 min (or 6 h). The retarding effect increased when Eudragit RS PO concentration was further increased from 27.26% *w*/*w* (F-7) to 41.03% *w*/*w* (F-8), where F-8 starts reaching 100% drug release after around 1440 min (or 24 h). It is worth pointing out that the influence of retardant concentration had more impact on the drug release rate than the water concentration, as seen when comparing F-7 and F-8. Despite the reduction of granulating liquid, which would usually lead to faster drug release due to the high propensity of disintegration, the increase in retardant had a more pronounced influence in reducing the drug release rate.

Overall, liquid vehicle does seem to have some influence on dissolution rate; however, it is the concentration of retardant that has the most noticeable effect. The best formulation (F-8) reaches about 100% drug release after 24 h. If 100% drug release is achievable after 24 h at acidic pH, then such a result would give matrix-based sustained-release Liqui-Tablet a similar dissolution performance as in a film-coated sustained-release propranolol hydrochloride dosage form [37]. With current data from this investigation, Liqui-Mass technology seems promising in delivering a sustained-release dosage.

### 3.7. Kinetic Model Analysis of Drug Release

The data from the in vitro dissolution test result was applied to different kinetic models and was evaluated by the square of correlation coefficient (*R*^2^) and mean percentage error (MPE). This is shown in Table 5, where data from the dissolution test were taken at pH 1.2 for the first 2 h then at pH 7.4 for an additional 22 h.

Since the mathematical model for the Higuchi and Korsmeyer–Peppas model are usually only valid for the first 60 % of the drug release [38], only zero-order and first-order models were applied to formulations with a fast drug release rate. The observation in the dissolution profiles (Figure 3) shows that most formulations have quick drug release, except for Liqui-Tablet with retarding agent (F-6 to F-8). Only zero-order and first-order mathematical models were applied to the quick drug releasing formulations. The results show that the release models for F-1 to F-5 follow the zero-order model, and the physical mixture tablet follows first-order model. This means that in formulation F-1 to F-5, the drug is released at a constant manner, which is beneficial in terms of maintaining a constant drug plasma level throughout the delivery. The physical mixture tablet follows the first-order kinetic, which means that the drug release for these formulations is dependent on concentration. The release is proportional to the amount of propranolol hydrochloride remaining in the dosage form; therefore, drug release diminishes over time [32]. It has been observed in a study from Mulye and Turco [39] that water-soluble drug in porous matrices follow the first-order model, which may suggest that the fast releasing tablets made in this investigation may be porous matrices. It is interesting that Liqui-Tablet follows a zero-order kinetic model.

When a sufficient amount of Eudragit RS PO is incorporated into Liqui-Tablet, it is clear that the kinetic model changes. The addition of the retarding agent at a concentration of 27.4% (F-7) and 41% (F-8) show the highest *R*^2^ value for the Higuchi model in Table 5 (*R*^2^ = 0.953 with MPE of 17.72, and *R*^2^ = 0.946 with MPE of 20.18, respectively). Based on the data from the Higuchi model, it can be interpreted that the key mechanism of drug release for F-7 and F-8 is diffusion-controlled [39].

Although the F-7 and F-8 drug release kinetic is predominantly Higuchi, the Korsmeyer–Peppas *R*^2^ values are equal to or above 0.914, and so there may be several release mechanisms involved. The *n* value for Korsmayer–Peppas in F-7 (*n* = 0.941) suggests case II transport and zero-order release [39]. Case II transport is considered to be an anomalous diffusion phenomenon that does not accord with Fick’s equation [40]. It is controlled by the swelling and relaxation of the drug delivery system matrix and is independent of time. As for F-8, the *n* value above 1 suggests super case II. This describes the influence of water uptake and swelling on the drug release for the polymeric and swellable system.

## 4. Conclusions

The study confirms that it is possible to produce an effective sustained release Liqui-Tablet using Eudragit RS PO within the matrix of the dosage form. The drug release of propranolol hydrochloride Liqui-Tablet was able to be sustained, with drug release reaching around 100% after about 24 h. The physical mixture tablet with Eudragit RS PO retarding agent did not have a very noticeable influence on retarding drug release. Liquid vehicles did display an influence on retarding drug release; however, when they were combined with Eudragit RS PO, there seems to be an obvious synergistic retarding effect, which can be effective at certain retardant concentrations. This suggests that Liqui-Mass technology may be an effective solution for producing sustained-release dosage in a simplistic and cost-effective manner. The drug release of the best formulation follows a Higuchi model, indicating a diffusion-controlled drug release. It was also found that liquid vehicle can significantly improve tablet hardness. In terms of pre-compressed Liqui-Tablet flow property, particle size distribution and Liqui-Tablet robustness, there seems to be no major issue.

## 5. Patents

Application no. PCT/GB2019/052065 was filed on 24 July 2019 and published internationally on 30 January 2020 (International Publication Number WO2020/021254 A1).

## Figures and Tables

**Table 1:** Key formulation characteristics of the investigated propranolol SR Liqui-Tablet and physical mixture tablet.

Formulation	Liquid Vehicle	Amount of Liquid Vehicle (% *w*/*w*)	Liquid Load Factor	Amount of Pre-Extrusion Liquid during Extrusion-Spheronization (mL) per 20 g of Admixture of API and Excipients	Mass of Avicel PH 102 (mg)	Mass of Aerosil 300 (mg)	Mass of Eudragit RS PO (mg)	Compression Force (PSI)	Total Weight of 80 mg Propranolol Liqui-Tablet (mg)
PMT-1 *	---	---	---	22.3	180	9	---	1000	269
PMT-2 **	---	---	---	14.9	135	9	45	1400	269
F-1	Tween 80	12.94	0.67	12.9	180	9	---	1400	309
F-2	Tween 20	12.94	0.67	12.9	180	9	---	1400	309
F-3	Kolliphor EL	12.94	0.67	12.9	180	9	---	1400	309
F-4	Tween 20	18.24	0.78	12.2	180	9	---	1400	329
F-5	Tween 20	22.92	0.89	11.5	180	9	---	1400	349
F-6	Tween 20	18.24	0.78	9.7	135	9	45	1400	329
F-7	Tween 20	18.24	0.78	4.86	90	9	90	1400	329
F-8	Tween 20	18.24	0.78	3.0	45	9	135	1400	329

Note all formulations contain 80 mg of propranolol HCl; water was used as pre-extrusion liquid, and the carrier to coating material was at a ratio of 20:1. * PMT-1, Physical mixture tablet without Eudragit RS PO. ** PMT-2, Physical mixture tablet with Eudragit RS PO.

**Table 2:** Solubility of propranolol hydrochloride in various solvents at 37 °C (*n* = 3).

Solvent	Mean Concentration (mg/mL) ± SD ^a^
Tween 80	1.89 ± 0.01
Tween 20	1.86 ± 0.00
Kolliphor EL	1.32 ± 0.00
Water	29.03 ± 0.12

^a^ For the composition of each formulation refer to Table 1.

**Table 3:** Flow rate (g/sec), angle of repose and Carr’s compressible index (CI%) of all formulations (*n* = 3).

Formulation ^a^	Flow Rate (g/sec) ± SD ^b^	Angle of Repose ± SD ^b^	CI% ± SD ^b^	Inference According to Angle of Repose	Inference According to CI%
PMT-1	6.86 ± 0.31	24.48 ± 1.17	16.17 ± 0.00	Excellent flowability	Fair flowability
PMT-2	8.31 ± 0.43	21.77 ± 0.21	14.26 ± 0.00	Excellent flowability	Good flowability
F-1	8.50 ± 0.14	20.77 ± 0.14	12.56 ± 2.06	Excellent flowability	Good flowability
F-2	8.17 ± 0.35	22.94 ± 0.65	13.53 ± 0.00	Excellent flowability	Good flowability
F-3	7.86 ± 0.33	22.79 ± 0.81	12.50 ± 0.00	Excellent flowability	Good flowability
F-4	6.50 ± 0.03	26.82 ± 0.88	13.86 ± 0.00	Excellent flowability	Good flowability
F-5	6.80 ± 0.32	23.39 ± 0.39	6.47 ± 0.00	Excellent flowability	Excellent flowability
F-6	6.44 ± 0.13	24.84 ± 1.14	12.79 ± 0.00	Excellent flowability	Good flowability
F-7	7.28 ± 0.21	24.11 ± 0.88	12.26 ± 0.00	Excellent flowability	Good flowability
F-8	7.04 ± 0.144	24.51 ± 0.74	11.84 ± 0.00	Excellent flowability	Good flowability

^a^ For the composition of each formulation refer to Table 1. ^b^ SD, standard deviation of the mean.

**Table 4:** Friability and tablet hardness test results of all formulations.

Formulation	% Weight Loss	Fractured (Yes/No)	Passed/Failed	Mean Hardness ± SD ^a^ (N)
PMT-1	---	Yes	Failed	17.67 ± 2.52
PMT-2	---	Yes	Failed	22.00 ± 3.61
F-1	0.00	No	Passed	103.00 ± 10.54
F-2	0.00	No	Passed	106.00 ± 3.61
F-3	0.08	No	Passed	111.33 ± 12.70
F-4	0.00	No	Passed	102.67 ± 6.43
F-5	---	Yes	Failed	95.67 ± 17.62
F-6	0.00	No	Passed	99.67 ± 10.02
F-7	0.00	No	Passed	69.33 ± 5.03
F-8	0.00	No	Passed	85.67 ± 4.04

^a^ SD, standard deviation of the mean.

**Table 5:** Release parameters of all formulations.

Formulation	Zero-Order Model *R*^2^		First-Order Model *R*^2^		Higuchi Model *R*^2^		Korsmeyer–Peppas Model			Best Fit Model
	*R* ^2^	MPE	*R* ^2^	MPE	*R* ^2^	MPE	*R* ^2^	MPE	*n*	
PMT-1	0.923	15.60	0.997	4.49	---	---	---	---	---	First-order
PMT-2	0.907	20.98	0.996	7.59	---	---	---	---	---	First-order
F-1	0.982	7.87	0.940	106.86	---	---	---	---	---	Zero-order
F-2	0.967	57.16	0.844	261.29	---	---	---	---	---	Zero-order
F-3	0.958	16.03	0.881	131.89	---	---	---	---	---	Zero-order
F-4	0.968	12.49	0.914	254.73	---	---	---	---	---	Zero-order
F-5	0.969	11.82	0.702	567.20	---	---	---	---	---	Zero-order
F-6	0.998	4.74	0.976	22.65	0.971	20.63	0.994	5.07	1.327	Zero-order
F-7	0.838	43.18	0.905	37.43	0.953	17.72	0.923	19.48	0.941	Higuchi
F-8	0.823	50.84	0.885	45.09	0.946	20.18	0.914	21.76	1.030	Higuchi

*R*^2^ is the square of correlation coefficient. *n* is the diffusional exponent or drug release exponent. MPE is the mean percentage error. --- due to fast drug release, these parameters cannot be calculated.

## Data Availability

Not applicable.
